# Filaggrin loss-of-function mutations and atopic dermatitis as risk factors for hand eczema in apprentice nurses: part II of a prospective cohort study

**DOI:** 10.1111/cod.12139

**Published:** 2013-09-19

**Authors:** Maaike J Visser, Maarten M Verberk, Linda E Campbell, W H Irwin McLean, Florentine Calkoen, Jan G Bakker, Frank J H van Dijk, Jan D Bos, Sanja Kezic

**Affiliations:** 1 Coronel Institute of Occupational Health, Academic Medical Centre, University of AmsterdamAmsterdam, Meibergdreef 15, 1105 AZ, The Netherlands; 2 Centre for Dermatology and Genetic Medicine, Colleges of Life Sciences and Medicine, Dentistry & Nursing, Medical Sciences Institute, University of DundeeDundee, Dow Street, DD1 5EH, UK; 3 Netherlands Centre for Occupational Diseases, Academic Medical Centre, University of AmsterdamAmsterdam, Meibergdreef 15, 1105 AZ, The Netherlands; 4 Department of Dermatology, Academic Medical Centre, University of AmsterdamAmsterdam, Meibergdreef 15, 1105 AZ, The Netherlands

**Keywords:** atopic dermatitis, filaggrin, hand eczema, nurses, occupational diseases, susceptibility

## Abstract

**Background/objectives:**

Environmental exposure and personal susceptibility both contribute to the development of hand eczema. In this study, we investigated the effect of loss-of-function mutations in the filaggrin gene (*FLG*), atopic dermatitis and wet work exposure on the development of hand eczema in apprentice nurses.

**Methods:**

Dutch apprentice nurses were genotyped for the four most common *FLG* mutations; atopic dermatitis and hand eczema history were assessed by questionnaire. Exposure and hand eczema during traineeships were assessed with diary cards.

**Results:**

The prevalence of hand eczema during traineeships was higher among subjects with a history of hand eczema reported at inclusion. Hand washing during traineeships and at home increased the risk of hand eczema. After adjustment for the effects of exposure and *FLG* mutations, an odds ratio of 2.5 (90% confidence interval 1.7–3.7) was found for a history of atopic dermatitis. In this study, an increased risk of hand eczema conferred by *FLG* mutations could not be shown, but subjects with concomitant *FLG* mutations and atopic dermatitis showed the highest risk of hand eczema during traineeships.

**Conclusion:**

A history of atopic dermatitis, a history of hand eczema and wet work exposure were the most important factors increasing the risk of hand eczema during traineeships.

Hand eczema (HE), as a manifestation of contact dermatitis of the hands, is one of the most common occupational diseases in industrialized countries, and may account for up to 90% of all occupational skin diseases 1,2. Skin exposure to irritants is a risk in occupations such as nursing, hairdressing, and metalworking; in these occupations, 1-year prevalence rates of HE of up to 30% have been reported 3–7. Although relevant exposure is a prerequisite for the development of occupational HE, some workers are more susceptible than others. The best-known and firmly established susceptibility factor for the development of occupational HE is a history of atopic dermatitis (AD). The increased risk of developing occupational HE for individuals with a history of AD has long been recognized 8–10, and recent population studies reported up to fivefold increased risks 11–13. One of the possible causes of the risk-enhancing effect of AD is an impaired skin barrier. Experimental studies have shown that the barrier function of the skin of patients with AD is reduced as compared with healthy controls, even in uninvolved skin areas 14–16. The mechanisms that underlie reduced skin barrier function in AD are not fully clear, but recent research suggests that the epidermal protein filaggrin might play an important role 17,18. In the stratum corneum, filaggrin contributes to structural strength by aggregating the keratin filaments, and its breakdown products support hydration, pH balance, anti-bacterial defence, and resistance to ultraviolet radiation 18,19. Several loss-of-function mutations have been identified in the filaggrin gene (*FLG*), resulting in reduced amounts or, in the case of homozygotes, in the absence of filaggrin in the skin. The summed prevalence of individuals who carry one or more of the most common *FLG* loss-of-function mutations in European populations is reported to be 7–10% 17,20–23. The impact of these mutations on skin barrier function has been shown in animal models 24, in patients with ichthyosis or AD 14,25, and in 3-month old infants with and without eczema 26. *FLG* loss-of-function mutations are strongly associated with AD; 16–44% of individuals with moderate to severe AD carry one or more *FLG* mutations 20,22,27–29. A recent meta-analysis revealed a more than threefold increased risk of developing AD in carriers of either one of the *R501X* or *2282del4* mutations 30. Because filaggrin is important for the barrier function of the skin, it is plausible that *FLG* mutations as such can increase the risk of occupational HE. Indeed, recent case–control studies found an association between *FLG* mutations and the occurrence of occupational HE 31–34. In the aetiological relationship between *FLG* mutations and occupational HE, AD can be both an intermediate factor (as *FLG* mutations increase the risk of AD) and a co-determinant independent from *FLG*. In the present study, we aimed to gain more insight into the relative contributions of both *FLG* mutations and AD to the aetiology of occupational HE.

Knowledge of susceptibility factors could contribute to more targeted prevention of occupational HE. In some countries, a history of HE and a history of AD are used to identify persons at risk in jobs with high skin exposure; susceptible workers are offered extra preventive measures and attention by their occupational physician 35,36. It has not yet been investigated whether the predictive value of susceptibility screening can be increased by adding a genetic susceptibility marker such as *FLG* mutations. Interestingly, 40% of *FLG* mutation carriers do not develop AD 19,37,38. This subgroup will not be recognized as susceptible in current prevention programmes. Another issue is that most of the studies that have explored the effect of *FLG* mutations on contact dermatitis 31–33,39–41 have not accounted for the extent of environmental exposure. Therefore, the relative contributions of *FLG* mutations and a history of or current AD, taking exposure into consideration, are still to be elucidated. We performed a prospective cohort study among apprentice nurses, who provided a DNA sample by buccal swab, filled in a questionnaire concerning symptoms of AD and atopy, and were consecutively followed up for 1–3 years, with regular monitoring of symptoms of HE as well as exposure to ‘wet work’ as assessed with diary cards. The general characteristics of this cohort, the exposure to wet work during follow-up and the occurrence of HE have been described in Part I of this study 42. The present article describes the influence of *FLG* mutations, AD and exposure on the risk of HE in this cohort.

## Methods

### Subjects

A detailed description of the study population and inclusion procedure is provided in Part I of this study 42. In short, apprentices were recruited from 15 different Dutch vocational schools that prepare students for a career in healthcare (nursing or care-giving). Students were eligible for participation if they had recently started a traineeship with a duration of at least 10 weeks, or were expecting to do so within the next few weeks. The only exclusion criterion was the presence of chronic inflammatory disease (e.g. psoriasis or rheumatoid arthritis). Approval was obtained from the Medical Ethical Committee of the Academic Medical Centre, Amsterdam, The Netherlands.

### DNA sampling and genotyping

The four most common *FLG* loss-of-function mutations in European populations were genotyped: *R501X*, *2282del4*, *R2447X*, and *S3247X*. Subjects provided a buccal swab sample (Geneticlab Diagnostic & Research, Pordenone, Italy; http://www.geneticlab.it), and DNA material was obtained from buccal mucosa cells. For each subject, two swabs were obtained, and 2 ml of lysis buffer (Puregene® Cell Lysis Solution; Gentra Systems, Minneapolis, MN, USA) was added to each swab to disrupt the cells and stabilize the DNA. Extraction and genotyping for the *FLG* mutations *R501X*, *R2447X* and *S3247X* was performed by KBioscience (http://www.kbioscience.co.uk). Genotyping was performed with the KASP single-nucleotide polymorphism genotyping system, a homogeneous fluorescence resonance energy transfer (FRET)-based system, coupled with competitive allele-specific polymerase chain reaction (PCR). Blind duplicates and Hardy–Weinberg equilibrium tests were used as quality control tests. *R501X* was genotyped by using the primer pair GAATGCCTGGAGCTGTCTCG (C-allele) and CTGAATGCCTGGAGCTGTCTCA (T-allele), with the common allele primer GCACTGGAGGAAGACAAGGATCG. *R2447X* was genotyped by using the primer pair GAGTGCCTGGAGCTGTCTCG (C-allele) and GAGTGCCTGGAGCTGTCTCA (T-allele), with the common allele primer GAGGAAGACAAGGATCCCACCACA. *S3247X* was genotyped by using the primer pair GTGTCTGGAGCCGTGCCTTG (C-allele) and GGTGTCTGGAGCCGTGCCTTT (A-allele), with the common primer CTTCCAGAAACCATCGTGGATCTGT. Genotyping for *2282del4* was performed by sizing a fluorescently labelled PCR fragment on an Applied Biosystems 3100 or 3730 DNA sequencer (Foster City, CA, USA), as described previously 32,43.

### Questionnaires

At inclusion, subjects filled in a questionnaire including items on eczema, rhinitis, conjunctivitis and asthma, allergies and/or symptoms following exposure to dust, animals, pollen, foods, metals, and wool, present or past skin diseases, the presence of any other chronic disease, medication use, present or past skin symptoms on the hands or fingers, and exposure to wet work during previous jobs/apprenticeships, secondary jobs, and leisure or household activities. Atopy was defined as the presence of two or more of the following: symptoms following exposure to common allergens, rhinitis, conjunctivitis, or asthma. AD was assessed according to a slightly modified version of the UK Working Party criteria ‘questions only’ definition, in which onset below 2 years of age was replaced by onset below 5 years of age as a proxy of ‘childhood dermatitis’.

At the end of the follow-up period, an email questionnaire was sent to all subjects still in the study. This final questionnaire included items on symptoms experienced during follow-up, consultation of general practitioners or dermatologists, changes in hand hygiene behaviour, the use of protective hand cream, information on traineeships and side jobs, and smoking.

### Exposure and symptoms during practical training

During their traineeships, the students had to keep count of the wet work activities that they performed during several shifts, using special diary cards as described in detail in Part I of this study 42. Skin symptoms on the hands were also recorded on the cards. If no cards had been returned near the end of the traineeship, students were contacted by email and/or telephone to retrieve information about the type of traineeship and possible symptoms retrospectively.

Following the classification for screening for HE symptoms proposed by Vermeulen et al. 44, HE was defined as the presence of at least one of the following symptoms of combinations: fissures and redness, fissures and itch, fissures and scaling, vesicles, or papules, plus duration of > 3 days or recurrence (symptoms reported more than once). As these criteria were originally developed for identifying cases of HE in a working population, we were concerned that, by using this definition, we would miss early-stage symptoms that may progress into HE. Therefore, we also used a more lenient definition, ‘mild HE’, defined as the presence of at least one of the following symptoms or combinations: redness and itch, redness and scaling, itch and scaling, fissures and redness, fissures and itch, fissures and scaling, vesicles, or papules, all irrespective of duration or recurrence.

Without specification, HE refers to any episode of HE during follow-up. Some analyses comprise students without a history of HE in contrast to students with a history of HE. The latter refers to HE at any time before or at inclusion. A first episode of HE during follow-up in a student without a history of HE is equivalent to ‘incident HE’.

### Statistical analysis

Statistical analysis was performed with ibm spss™ statistics version 19 and Microsoft Excel™. In the subgroup analyses, *FLG* mutation carriers were compared with *FLG* wild-type individuals. No distinction was made between homozygous, compound heterozygous or heterozygous *FLG* mutation carriers, because the subgroup of homozygous or compound heterozygous carriers was too small for subgroup analysis to be performed. Because AD can be both an intermediate factor and an independent co-determinant in the aetiological relationship between *FLG* mutations and HE, we chose to use stratified analyses to study the effects on HE of *FLG* mutations and AD, each in the absence and in the presence of the other factor.

The relative risks (RRs) and confidence intervals (CIs) for the subgroup analyses of HE symptoms reported at inclusion were computed by using cross-tabulated results and applying the following formulas in Excel: RR = [*a*/(*a* + *b*)]/[*c*/(*c* + *d*)], with *a* and *c* being the numbers of HE cases in the ‘exposed’ and ‘referent’ groups, respectively; and 90% CI(RR) = exp{ln(RR) ± 1.645*SE[ln(RR)]}, in which SE[ln(RR)] = sqrt{*b*/[*a*(*a* + *b*)] + *d*/[*c*(*c* + *d*)]}. The 90% CI corresponds to one-sided testing with *p* < 0.05.

Analysis of the combined influence of susceptibility and exposure factors on the risk of HE during follow-up was performed with generalized linear mixed models in spss. HE is a disease with a fluctuating course, and the recovery time may be as short as a few days. Thus, the apprentice nurses would have time to recover from HE in between traineeship periods. We therefore assumed that the probability of developing HE in one traineeship does not depend on the extent of exposure or on having had HE in previous traineeships. Each traineeship was therefore counted as a separate entity, and data from subjects who entered a second or a third traineeship were entered as multiple records in the database. This, however, results in the problem that, regardless of susceptibility, subjects who contributed data for multiple traineeships would have had more opportunities to develop HE than those who had been followed for only one traineeship. Therefore, a mixed models design was used in the analyses, with participant ID included as random effect (procedure GENLINMIXED in spss™). In such a mixed model, the within-subject correlation is taken into account.

Analysis of wet work exposure in this cohort (Part I) had shown that a frequency of hand washing during practical training of > 8 times per shift, hand washing at home > 10 times per day and working in a side job involving wet work for > 8 hr a week increased the risk of HE (Part I) 42. Therefore, these were included as binary variables in the multivariate mixed models to represent wet work exposure. Preceding this analysis, the mean frequency of hand washing in different healthcare sectors was assessed with linear mixed models with healthcare sector as fixed effect and subject identification as random effect (procedure MIXED in spss™). The mean frequency of hand washing during traineeships was lowest in psychiatry (7.0 times per shift), medium to high in homecare and hospitals (8.8 and 8.9 times per shift, respectively), highest in care for the disabled and nursing homes (10.4 and 10.5 times per shift, respectively). The frequency of hand washing during traineeships was classified according to whether the traineeship was performed in psychiatry or in any other sector, which corresponds to a cut-off value of (supposedly) 8 times per shift. This classification was applied to all subjects.

Use of hand cream, exposure to wet work and number of subjects reporting HE during traineeships (Fig. 2) was compared between the four subgroups categorized by *FLG* and AD by use of the chi-square test.

**Figure 1:**
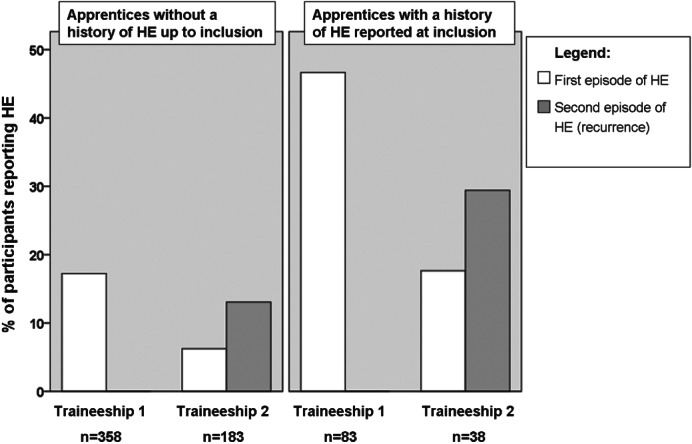
Reported period prevalence of hand eczema (HE) during traineeships in the first and second years of follow-up in subjects with or without a history of HE reported at inclusion.

**Figure 2:**
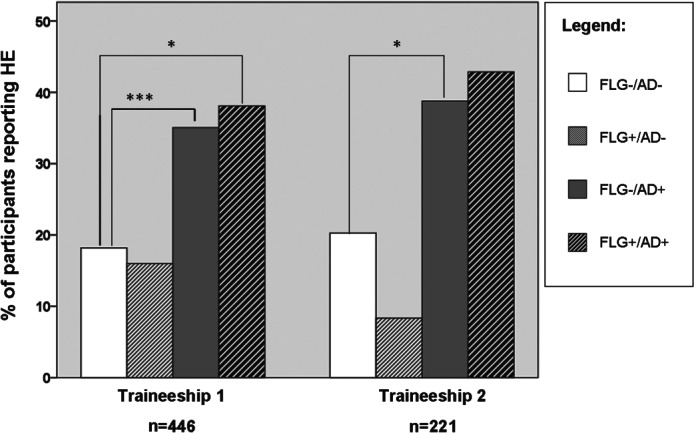
Reported period prevalence of hand eczema (HE) during the first and second traineeships in four subgroups of participants. *p < 0.05; ***p < 0.0001.

## Results

### Study population

The participation rate of the apprentices invited was ˜ 50%. A total of 728 apprentice nurses completed the inclusion questionnaire. Seven apprentices were excluded because of chronic inflammatory disease. Some subjects did not provide a buccal swab sample at inclusion, because they were aged < 18 years (in The Netherlands, these persons are only allowed to provide a DNA sample with parental consent), and DNA sampling was postponed until parental consent was obtained or until they had turned 18 years during follow-up. Eventually, a total of 626 DNA samples were obtained, 596 of which were successfully genotyped for all four investigated *FLG* loss-of-function mutations (*R501X*, *2282del4*, *R2447X*, and *S3247X*). A further 150 subjects were lost to follow-up shortly after completion of the inclusion questionnaire or quit the study before going through a traineeship (mostly because of changing career), leaving a total of 446 subjects in whom to study the impact of susceptibility factors on the risk of developing HE during vocational training.

### Genotype distributions and associations with atopic disease

Table1 shows the genotype distributions. *FLG* mutations were present in 11.1% of the subjects. Fifty-six individuals were heterozygous for one mutation, 2 were homozygous for *2282del4*, 3 were homozygous for *R2447X*, and 1 was compound heterozygous for *2282del4* and *R501X*.

**Table 1:** Genotype distributions for the filaggrin gene *FLG* loss-of-function mutations *R501X, 2282del4, R2447X* and *S3247X* in apprentice nurses

*FLG* mutation	*R501X*	*2282del4*	*R2447X*	*S3247X*	Combined (four mutations)
Group size	608	614	610	607	596
AA, n (%)	587 (96.5)	576 (93.8)	604 (99.0)	605 (99.7)	530 (88.9)
Aa, n (%)	21 (3.5)	35 (5.7)	3 (0.5)	2 (0.3)	59 (9.9)
aa, n (%)	0 (0)	3 (0.5)	3 (0.5)	0 (0)	7 (1.2)
Total *FLG* carriers (Aa + aa), n (%)	21 (3.5)	38 (6.2)	6 (1.0)	2 (0.3)	66 (11.1)
Wild-type allele frequency (%)	98.3	96.7	99.3	99.8	93.9
Mutant allele frequency (%)	1.7	3.3	0.7	0.2	6.1

The genotype distributions were not in Hardy–Weinberg equilibrium for *2282del4*, *R2447X*, and the combined genotype. This was probably because of the relatively large number of homozygotes among subjects with AD, combined with a slight overrepresentation of subjects with AD in this cohort (see Part I) 42. In subjects without AD (n = 460), the genotype distributions for *2282del4* and the combined genotype were in Hardy–Weinberg equilibrium.

*FLG* mutations were associated with a history of AD (RR 1.8; 90% CI 1.37–2.35), especially with persistent AD starting before 5 years of age and still present at the time of inclusion (RR 2.6; 90% CI 1.43–4.67). There were also associations between *FLG* mutations and general dry skin (RR 2.5; 90% CI 1.82–3.38) and between *FLG* mutations and symptoms upon exposure to common allergens (RR 1.3; 90% CI 1.01–1.54). No association was found with rhinitis or asthma.

### Symptoms of HE reported at inclusion

We used stratified analyses to investigate the effect of *FLG* mutations and AD on the occurrence of HE. The study population was divided into four groups: (i) subjects without *FLG* mutations and with no history of AD (*FLG−*/AD*−*); (ii) subjects with *FLG* mutations but with no history of AD (*FLG+*/AD*−*); (iii) subjects without *FLG* mutations but with a history of AD (*FLG−*/AD+); and (iv) subjects with both *FLG* mutations and a history of AD (*FLG+*/AD+). In a retrospective approach, we compared past and present symptoms of HE reported in the inclusion questionnaire between these four groups (Table2). In total, 54% of all subjects reported one or more skin symptoms, 13% had a history of HE, and 7% had HE at the time of inclusion. Regardless of *FLG* mutations, a history of AD conferred an increased RR for all investigated symptoms. Subjects with concomitant *FLG* mutations and AD (*FLG+*/AD+) showed the highest symptom prevalence, and a significantly higher prevalence of scaling, fissures and current HE than the *FLG−*/AD+ subgroup. Among subjects without a history of AD, those who carried one or more *FLG* mutations (*FLG*+/AD*−*) did not report more symptoms on the hands and fingers or HE before or at inclusion than those without *FLG* mutations.

**Table 2:** Number of apprentice nurses reporting having (or having had) symptoms of hand eczema at inclusion, and relative risk (RR) ratios for symptoms of hand eczema in four subgroups based on filaggrin gene *FLG* mutations and history of atopic dermatitis (AD)

Subgroup name:	*FLG−*/AD*−*	*FLG*+/AD*−*	*FLG−*/AD+	*FLG*+/AD+	RR	RR	RR	RR	RR
Subgroup characteristics	(Reference)								
FLG mutations^a^	No	Yes	No	Yes	*FLG*+/AD*−*	*FLG−*/AD+	*FLG*+/AD+	*FLG*+/AD+	*FLG*+/AD+
History of AD^b^	No N = 405	No N = 38	Yes N = 125	Yes N = 28	versus *FLG−*/AD*−*	versus *FLG−*/AD*−*	versus *FLG−*/AD*−*	versus *FLG*+/AD*−*	versus *FLG−*/AD+
Reported symptoms on the hands/fingers at inclusion	n (%)	n (%)	n (%)	n (%)	RR (90% CI)	RR (90% CI)	RR (90% CI)	RR (90% CI)	RR (90% CI)
Redness	84 (21)	7 (18)	60 (48)	16 (57)	0.9 (0.50–1.59)	2.3* (1.85–2.89)	2.8* (2.01–3.77)	3.1* (1.48–6.51)	1.2 (0.87–1.62)
Scaling	88 (22)	5 (13)	47 (38)	15 (54)	0.6 (0.30–1.22)	1.7* (1.35–2.21)	2.5* (1.78–3.42)	4.1* (1.68–9.88)	1.4* (1.01–2.01)
Itching	145 (36)	14 (37)	74 (59)	18 (64)	1.0 (0.71–1.48)	1.7* (1.40–1.95)	1.8* (1.39–2.32)	1.7* (1.06–2.88)	1.1 (0.84–1.41)
Fissures	91 (22)	11 (29)	46 (37)	15 (54)	1.3 (0.83–2.01)	1.6* (1.28–2.09)	2.4* (1.72–3.31)	1.9* (1.01–3.39)	1.5* (1.03–2.06)
Vesicles	48 (12)	0 (0)	32 (26)	8 (29)	—	2.2* (1.54–3.02)	2.4* (1.41–4.14)	n.a.	1.1 (0.64–1.94)
Bumps	74 (18)	2 (5)	46 (37)	9 (32)	0.3* (0.09–0.91)	2.0* (1.55–2.61)	1.8* (1.08–2.85)	6.1* (1.43–26.10)	0.9 (0.53–1.43)
History of mild hand eczema^c^	163 (40)	13 (34)	81 (65)	19 (68)	0.9 (0.58–1.25)	1.6* (1.39–1.87)	1.7* (1.33–2.13)	2.0* (1.19–3.30)	1.1 (0.82–1.33)
History of hand eczema^d^	54 (13)	2 (5)	37 (30)	12 (43)	0.4 (0.12–1.25)	2.2* (1.63–3.02)	3.2* (2.12–4.87)	8.1* (1.98–33.52)	1.5 (0.95–2.21)
Mild hand eczema currently present	58 (14)	7 (18)	32 (26)	9 (32)	1.3 (0.71–2.33)	1.8* (1.30–2.46)	2.2* (1.37–3.68)	1.7 (0.74–4.12)	1.3 (0.75–2.10)
Hand eczema currently present	18 (4)	1 (3)	14 (11)	7 (25)	0.6 (0.11–3.14)	2.5* (1.44–4.42)	5.6* (2.91–10.87)	9.5* (1.24–72.89)	2.2* (1.13–4.40)

CI, confidence interval.

a Carrier of one or more of the following loss-of-function mutations in *FLG*: *R501X*, *2282del4*, *R2447X*, or *S3247X*.

b A history of AD was assessed by questionnaire, with a slightly modified version of the UK Working Party criteria.

c One or more of the following combinations of symptoms: redness and itch, redness and scaling, scaling and itch, fissures and redness, fissures and itch, fissures and scaling, vesicles, or papules.

d One or more of the following combinations of symptoms: fissures and redness, fissures and itch, fissures and scaling, vesicles, or papules, plus duration of > 3 days or recurrence (symptoms reported more than once).

* Significant at *α* < 0.05.

Of the 7 homozygous or compound heterozygous subjects among the *FLG* mutation carriers, 6 had a history of AD, 3 had HE at the time of inclusion, and another 3 reported a history of HE.

### HE during traineeships: effects of AD, *FLG* mutations, and wet work

One to three years of follow-up was completed for 446 subjects. One hundred and thirty subjects (29%) reported HE on one or more occasions during their traineeships. Three hundred and fifty-nine subjects (81%) had no HE history up to the time of inclusion. Of these, 78 (22%) developed HE during their traineeships. Among the subjects with a history of HE but no HE at the time of inclusion (n = 52), 29 (56%) reported HE during one or more traineeships. Thirty-five subjects (8%) had HE at the time of inclusion. Mixed models analysis showed that, after adjustment for the effects of exposure, subjects with a history of HE up to inclusion were at increased risk of developing HE during traineeships [odds ratio (OR) 4.5; 90% CI 2.96–6.98]. After taking into account AD history, the OR for having HE during traineeships for subjects with a history of HE was 3.9 (90% CI 2.5–6.1). Twenty subjects who had HE at the time of inclusion also reported HE during their first traineeship. Because, for these subjects, it was unknown whether their HE was related to their traineeship or was a continuation of already existing HE, a second analysis was performed excluding these subjects, which resulted in an OR of 2.9 (90% CI 1.80–4.70). Figure 1 shows the prevalence of HE in subjects with or without a history of HE reported at inclusion, divided into first and recurrent episodes of HE; it shows the high prevalence of HE during traineeships among subjects with a history of HE at inclusion and the high recurrence rate for this group.

The prevalence of HE during the first and second traineeships in the four subgroups of subjects with or without AD and *FLG* mutations is shown in Fig.2. Increased prevalence rates of HE were seen for the *FLG−*/AD+ subgroup and the *FLG+*/AD+ subgroup as compared with the *FLG−*/AD*−* control group. Both Figs.1 and 2 are restricted to the first and second traineeships, because the number of subjects who had completed a third traineeship was too small (n = 57) for subgroup analysis to be performed.

The exposure did not differ appreciably between the four subgroups; the proportion of subjects who had traineeships in healthcare sectors with frequent hand washing (cut-off at > 8 times per shift) ranged from 63% to 71%.

The effects of AD, *FLG* mutations and exposure on the risk of developing HE during traineeships were calculated with a mixed models design. On the basis of the results of Part I of this study 42, the frequency of hand washing during traineeships, hand washing at home > 10 times a day and working in a side job involving wet work (e.g. healthcare, bars, or restaurants) were included in the models to account for wet work exposure. A first crude analysis resulted in an unadjusted OR of 1.1 (90% CI 0.7–2.0) for *FLG* mutations and an unadjusted OR of 2.8 (90% CI 1.9–4.1) for AD.

Table3 shows the results of two multivariate mixed models including the four susceptibility subgroups together with exposure; in both models, the occurrence of mild HE in *FLG* wild-type subjects without AD serves as the reference. Model 1 shows that, after adjustment for the effects of exposure, a history of AD and the combination of a history of AD and *FLG* mutations increased the risk of HE during vocational training with ORs, respectively, of 2.2 and 3.6. Taking into account the group sizes, the weighted OR of AD was 2.5 (90% CI 1.7–3.7). For mild HE, the corresponding ORs were both 2.1. For *FLG* mutations in subjects without a history of AD, no effect could be shown (OR 0.7). Frequent hand washing during traineeships (> 8 times per shift) and frequent hand washing at home (> 10 times per day) increased the risk of hand eczema, with ORs of 2.2 and 1.8, respectively.

**Table 3:** Multivariate mixed models including atopic dermatitis (AD), filaggrin gene *FLG* loss-of-function mutations and exposure to frequent hand washing as risk factors for hand eczema (HE) during traineeships

		Model 1		Model 2
		Mild HE during practical training	HE during practical training	HE during practical training in subjects without previous wet work exposure and with no history of HE up to inclusion
No. of subjects included	—	448	448	247
No. of exposure records	—	667	667	375
Factor	—	OR (expβ) (90% CI)	OR (expβ) (90% CI)	OR (expβ) (90% CI)
*FLG* mutations and AD	*FLG*: no AD: no	1.0 (reference)	1.0 (reference)	1.0 (reference)
	*FLG*: yes AD: no	0.9 (0.4–1.8)	0.7 (0.3–1.7)	0.5 (0.1–1.9)
	*FLG*: no AD: yes	2.1 (1.0–3.1)*	2.2 (1.4–3.4)*	1.4 (0.7–2.9)
	*FLG*: yes AD: yes	2.1 (1.0–4.0)*	3.6 (1.7–7.5)*	3.7 (1.0–13.5)
Frequent hand washing during traineeships (> 8 times per shift)^a^	Yes versus no	1.4 (0.9–2.3)	2.2 (1.2–4.2)*	1.4 (0.6–3.4)
Frequent hand washing at home (> 10 times per day)	Yes versus no	1.8 (1.2–2.8)*	1.8 (1.1–2.9)*	1.9 (0.9–4.2)
Working in a side job involving wet work for > 8 hr/week	Yes versus no	1.6 (1.2–2.3)*	1.0 (0.7–1.5)	1.2 (0.6–2.1)

CI, confidence interval; OR, odds ratio.

a Based on the healthcare sector means of reported frequency of hand washing on exposure cards.

* *p* < 0.05.

Model 2 concerns HE during traineeships in subjects who had been free from HE up to inclusion, and who had not been exposed to skin irritants before entering the study (e.g. previous education or career involving wet work or traineeships in previous school years, because the extent of that exposure could not be estimated). A similar tendency for an increased risk of HE was found for AD in combination with *FLG* mutations and for frequent hand washing at home.

As these results suggest that the influence of *FLG* mutations on HE differs between subjects with and without AD, we investigated the existence of interaction in a model including *FLG*, AD, an interaction term between *FLG* and AD, and exposure. In the model including all participants (Model 1), no significant interaction effect could be shown [OR (interaction) 2.1; 90% CI 0.6–7.1]. In the model including only subjects without previous HE or exposure (Model 2), a tendency (*p* = 0.08) for interaction was found [OR (interaction) 5.4; 90% CI 1.1–25.9].

### Use of hand cream

Use of hand cream at least once a day was reported by 53% of the subjects in the *FLG−*/AD*−* subgroup. The use of hand cream was significantly more frequent than this in the *FLG−*/AD+ subgroup (68%, chi-square, *p* = 0.04) and in the *FLG+*/AD+ subgroup (90%, *p* = 0.006), but not in the *FLG*+/AD*−* subgroup (62%, *p* = 0.190).

## Discussion

This study examined both genetic susceptibility and environmental exposure as risk factors for HE. Regarding AD, we found a distinct effect on HE with both the follow-up and the retrospective approaches. During follow-up, we found no indication of an increased risk of HE conferred by *FLG* loss-of-function mutations, although the subjects with *FLG* mutations in addition to a history of AD had the highest OR for HE during traineeships (OR 3.6). With the retrospective approach, *FLG* mutations only had an effect on HE at inclusion in subjects with AD. Frequent hand washing during traineeships (> 8 times per shift) or at home (> 10 times per day) increased the risk of HE during follow-up, with ORs of 2.2 and 1.8, respectively.

Our results confirm that a history of AD is associated with an increased risk of HE in high-risk occupations, a finding that has been made in several epidemiological studies 45–49.

The fact that we could not show an effect of *FLG* mutations in the present study was unexpected. The high OR for HE during traineeships among subjects with concomitant *FLG* mutations and AD is consistent with the results of our recent case–control study on occupational contact dermatitis patients and vocational students in training for high-risk occupations. In that study, an effect of *FLG* mutations irrespective of AD (OR 1.6; 95% CI 1.0–2.6) was found 34. The cases in that study, however, had chronic and severe HE, as opposed to the apprentice nurses, who often had less severe HE, which might not become chronic. Possibly, higher exposure or a longer duration is needed to reveal an effect of *FLG* mutations.

In a recent cross-sectional population study, Thyssen et al. found that *FLG* mutations constituted a risk factor for HE in individuals with AD, but not in individuals without AD 33. Our data also point to an interactive effect, although this was only indicated in part of the analyses (Model 2).

The absence of a significant effect of *FLG* mutations in this study could not be explained by differences in exposure or use of hand cream. A possible explanation might be that that some *FLG* mutation carriers are able to compensate for reduced amounts of filaggrin in their skin via an as yet unknown mechanism, preventing them from developing AD as well as HE. This may partly explain the wide range in susceptibility to HE (OR 0.7–3.6) that we observed among *FLG* mutation carriers, which – in view of the observed tendency for interaction – is partly related to the absence or presence of AD. More research into the skin barrier properties of *FLG* mutation carriers without AD may shed more light on this.

Irrespective of *FLG* mutations, a possible role of the filaggrin protein itself may be considered. Recently, one study investigated skin lipid composition, irritation response and the skin barrier in AD patients and controls, both with and without *FLG* mutations. No differences in stratum corneum lipid composition or increases in transepidermal water loss after a 24-hr irritation test were found between *FLG* mutation carriers and *FLG* wild-type individuals 50. Another recent study also found no difference in lipid composition and skin barrier function between AD patients with and without *FLG* mutations. However, there was a significant positive correlation between favourable lipid organization and skin barrier function and natural moisturizing factors (NMFs) in the stratum corneum 51. As NMFs can be seen as a proxy for filaggrin expression 52,53, this might imply that filaggrin itself does play a role in the stratum corneum lipid composition and skin barrier function. Indeed, research among ichthyosis vulgaris patients carrying *FLG* mutations showed that filaggrin deficiency led to a paracellular defect in skin barrier function, caused by disrupted lipid bilayer organization and altered loading of lamellar bodies 25. In addition to the loss-of-function mutations, several other factors, mostly associated with AD, can influence filaggrin levels in the skin. For instance, the expression of filaggrin may be downregulated by inflammatory cytokines, for example interleukin (IL)-4, IL-13, IL-22, and IL-25 37,54–56. Also, Brown et al. have recently shown that the number of filaggrin repeats in the *FLG* gene may vary between 10, 11 and 12, and that these copy number variations are significantly associated with the risk of AD 57. It might be speculated that variation in filaggrin expression caused by copy number variations may also play a role in susceptibility to occupational HE. Future studies investigating the role of filaggrin in occupational HE should consider the inclusion of copy number variations in their analysis.

Some limitations of this study need to be mentioned. First, because of the multiple traineeships in which participants were repeatedly at risk of HE, a mixed model analysis was used. We note that the ORs obtained from the models are an overestimation of the RRs. This especially applies to subgroups with a high prevalence of HE. Corresponding RRs can be calculated by using the estimated means obtained from the models. For example, in Model 1, the ORs for HE of 0.7, 2.2 and 3.6 for the *FLG+*/AD*−*, *FLG−*/AD+ and *FLG+*/AD+ subgroups correspond to RRs of, respectively, 0.7, 1.9, and 2.5.

Second, detailed information on wet work exposure was available for only 383 of the 446 subjects who were followed up in this cohort. Repeating the mixed models analysis in this subset of subjects yielded similar results as when all subjects were included, which justifies the use of extrapolated exposure variables.

A third limitation is that, on the basis of the symptoms as reported, we were not able to distinguish between HE of the irritant, allergic or atopic type. Despite a similar clinical appearance, these subtypes of HE have different underlying mechanisms, and are probably not equally affected by genetic susceptibility factors. For example, *FLG* loss-of-function mutations have shown positive associations with irritant dermatitis 32,40, but less so with allergic contact dermatitis 31,39. Patch testing would be needed to differentiate between subjects with irritant HE and those with allergic HE, but this was not feasible in our study. Among the 52 subjects with reported symptoms of HE who were seen by the collaborating occupational physician, contact allergy was diagnosed in, at most, 23% (Part I) 42. If we had been able to exclude the cases with contact allergy from our study, this would probably have shifted the ORs for HE resulting from *FLG* mutations a little away from 1. Also, we were not able to assess severity of HE on the basis of the self-reported symptoms. The use of hand cream by 90% of the subjects in the *FLG+*/AD+ subgroup, however, suggests more severe HE in this subgroup. Possibly, a stronger effect of *FLG* mutations would have been found in association with severity of HE.

One of the underlying reasons for this study was to investigate whether adding *FLG* genotyping to the AD screening tool would improve the identification of susceptible individuals in high-risk occupations. Our results do not convincingly indicate that this is the case. Even if the effect of *FLG* mutations in subjects with a history of AD had been significant, the effect size would probably be too small for a substantial favourable effect on the predictive values of a screening procedure. Information about AD and HE history, as is currently asked for according to Dutch and German guidelines, is a feasible predictor for the acquisition of occupational HE, as our results have confirmed. The results of our case–control study 34 and our present prospective cohort study show that those in the *FLG+*/AD+ subgroup are at the highest risk for ocupational HE. Furthermore, occupational HE patients with concomitant AD and *FLG* mutations appeared to have a worse prognosis than *FLG−*/AD*−*, *FLG−*/AD+ or *FLG+*/AD*−* patients in a recent follow-up study 58. New research might confirm that AD patients with *FLG* mutations are indeed substantially more susceptible to occupational HE than patients with AD without *FLG* mutations. If this is so, identifying *FLG* mutations among AD patients and advising avoidance of irritant exposure in such patients would be beneficial.

## Conclusion and Recommendations

A history of AD, a history of HE and wet work exposure were the most important factors increasing the risk of HE during traineeships. As our results confirm that HE develops shortly after the start of exposure to wet work, even in traineeships, it is strongly recommended to start prevention programmes as early as during vocational training, instead of at the time of employment. In addition, it would be interesting to further investigate the skin barrier properties of *FLG* mutation carriers without AD, which may shed more light on the existence of possible mechanisms to compensate for reduced filaggrin in the skin.
